# Association Between Toothbrushing and Behavioral Risk Factors of Non-communicable Diseases: A population Based Survey of 4500 adults in China

**DOI:** 10.1038/s41598-019-44662-w

**Published:** 2019-06-11

**Authors:** Wenzhao Liu, Lingyu Su, Xudong Xie, Xuerong Xiang, Jiao Huang, Ping Ji

**Affiliations:** 1 Department of Periodontics, College of Stomatology, Chongqing Medical University, Chongqing, China; 2 Department of Maxillofacial Surgery, College of Stomatology, Chongqing Medical University, Chongqing, China; 3 Chongqing Key Laboratory for Oral Diseases and Biomedical Science, Chongqing, China; 4 Chongqing Municipal Key Laboratory of Oral Biomedical Engineering of Higher Education, Chongqing, China

**Keywords:** Tooth brushing, Epidemiology, Epidemiology, Risk factors

## Abstract

Non-communicable Disease (NCD) related behavioral risk factors (BRF) plays a crucial role in NCD prevention, as does oral hygiene behavior in oral health promotion. We examined the association between NCD BRF and toothbrushing using data from a population-based survey, which recruited 4485 adults aged 18+ years, in Chongqing city, China. Prevalence of five NDC BRF and their clustering within individual were determined by toothbrushing frequency. Ordinal logistic regression examined the association between toothbrushing and BRF clustering. Prevalence of current smoking, insufficient intake of vegetable and fruit, and harmful use of alcohol increased significantly with toothbrushing frequency. Respondents who brushed teeth ≥2 times daily consumed more red meat than those with less frequent toothbrushing. Relative to those with no BRF, the adjusted cumulative odds ratio of brushing teeth less frequently was 2.1 (95% CI: 1.4–3.1) for respondents with 3+ BRF. The adjusted cumulative odds ratio was 1.5 (1.1–2.1) and 1.4 (1.0–1.8) for those who had two BRF and those who had one, respectively. Significant correlation between toothbrushing and NCD BRF implied that integrated intervention strategy involving the both may be beneficial in public health programs targeting at either oral health or NCDs, or both.

## Introduction

Non-communicable diseases (NCDs) have been the top killers of Chinese population, accounting for 86.7% of total deaths in 2013^1^. Periodontal disease, one of the most common chronic inflammatory diseases, may place individuals at increased risk of developing NCDs, such as diabetes^2–4^, cardiovascular disease^5–7^, and adverse pregnancy^8^. The 4th National Oral Health Survey conducted in 2015 to 2016 reported that the prevalence of health periodontal condition for Chinese adults in age group of 35–44 years, 55–64 years, and 65–74 years was only 9.1%, 5.0%, and 9.3%, respectively^9^. Poor oral hygiene is recognized as the most important cause for periodontal disease^10^. Poor oral hygiene could cause plaque and calculus accumulation around teeth and result in inflammation of gingival tissues. Though reversible, gingivitis may progress to periodontitis without appropriate oral hygiene. Adequate oral hygiene also plays an important role in secondary prevention of periodontitis aiming to avoid disease recurrence in patients who have been successfully treated^11^. The most predictable indicator of oral hygiene is toothbrushing frequency. Toothbrushing serves as an effective and easily adjustable behavior for prevention of periodontal disease. It was reported as well that low toothbrushing frequency increased the risk of cardiovascular disease event^12^.

A Population-based primary prevention through modifying the major behavioral risk factors (BRF) is an essential strategy to address up-soaring disease burden of NCDs^13^. Some NCDs related BRF also contribute to the development of oral diseases, such as smoking^14^ and alcohol drinking^15^. From this viewpoint, oral diseases appear to be associated with NCDs as a result of shared risk factors. Understanding the correlations or clustering among those shared risk factor may help inform policy makers when tailoring public health strategy targeting both NCDs and oral diseases.

Previous researches in China have well documented the correlations between NCDs related BRF and associated factors with clustering of BRF^16,17^, but few studies looked into mutual correlations between oral health behavior and NCDs related BRF. The present study analyses data from a population-based survey in China, in order to clarify the relationship between toothbrushing and NCD related BRF, while controlling for other potential confounders.

## Results

### Basic characteristics of the study sample

Table 1 presents characteristics of the study sample. Overall, respondents had an average (SD) age of 57.9 (13.1) years, and 38.3% of them were men. Respondents with higher toothbrushing frequency were younger than were those with lower frequency. Men appeared to have poorer oral hygiene practice than did women. Only 33.2% of respondents who brushed their teeth at least twice per day were men, while the percentage was much higher (54.2%) in those who rarely or never brushed teeth. Compared to higher toothbrushing frequency, respondents who barely or never brushed teeth tended to have less favorable socioeconomic status. For example, they lived in a house with the lowest level of average annual family income ($1093), 59.2% of them received no education, and 26.6% lived in urban areas. In contrast, respondents brushing teeth at least twice a day had much higher average family income of $2624, only 24.1% of them received no education, and 65.7% lived in urban areas. The mean (SD) BMI was 24.0 (3.2) kg/m^2^ for respondents with toothbrushing frequency of ≥ twice a day, 24.2 (3.4) kg/m^2^ for once a day, and 23.2 (3.2) kg/m^2^ for rarely or never. Among respondents who barely or never brushed teeth, 22.3% reported they had been diagnosed with hypertension, and the percentage was 19.1% in both other two groups with higher toothbrushing frequency. Three-point nine percent of respondents who barely or never brushed teeth reported diagnosed diabetes, lower than those brushed teeth at least twice a day (7.4%) and those brushed once a day (4.5%).

**Table 1:** Characteristics of survey participants by tooth brushing frequency.

	At least twice a day (n = 1101)	Once a day (n = 2403)	Rarely or never (n = 961)	Total (n = 4465)
Mean age (SD)–years	54.5 (14.8)	57.1 (12.1)	64 (11.6)	57.9 (13.1)
Male sex–%	33.2	34.3	54.2	38.3
No education–%	24.1	42.3	59.2	41.5
Living in couples–%	17.5	13.8	22.3	16.5
Employed–%	63.7	74.4	83.4	73.7
Median annual household income per capita (IQR)- US$*	2624 (2624)	1640 (2132)	1093 (1397)	2624 (2329)
Living in urban areas–%	65.7	41.9	26.6	44.5
BMI (SD)–kg/m^2^	24 (3.2)	24.2 (3.4)	23.2 (3.2)	23.9 (3.3)
Self-reported hypertension-%	19.1	19.1	22.3	19.8
Self-reported Diabetes-%	7.4	4.5	3.9	5.1

*Based on the exchange rate of 6.3 renminbi to US$ 1 that was in effect on 30 September 2012.

### BRF prevalence

BRF prevalence by toothbrushing frequency were shown in Table 2. Current smoking (31.2%, 95% CI: 27.9–34.4%), insufficient intake of vegetable and fruit (46.5%, 43.1–49.9%), and high consumption of red meat (46.4%, 43.2–50.0%) prevailed at a high level, while harmful use of alcohol (9.7%, 7.8–11.7%) and physical inactivity (11.9%, 9.9–14.0%) were not rarely seen in the population. Prevalence of current smoking, insufficient intake of vegetable and fruit, and harmful use of alcohol increased significantly with toothbrushing frequency (P for trend <0.05). On the contrary, respondents who brushed teeth ≥2 times a day consumed more red meat than their counterparts with less frequent toothbrushing. No significant difference was observed in the prevalence of physical inactivity across toothbrushing frequency. We found a significant correlation between number of BRF and toothbrushing frequency: BRF tended to cluster within respondents who brushed teeth less frequently (P for correlation <0.05).

**Table 2:** Prevalence of NCD Related Behavioral Risk Factors by Tooth Brushing Frequency and residency areas*.

	Toothbrushing frequency			Total
	At least twice a day	Once a day	Rarely or never	
**Risk factor**				
Current smoking^†,‡^	24.1 (18.1,30.0)	29.2 (24.7,33.8)	52.0 (46.4,57.7)	31.2 (27.9,34.4)
Insufficient intake of vegetable and fruit^§,‡^	42.3 (35.1,49.6)	46.2 (41.6,50.8)	56.1 (50.5,61.7)	46.5 (43.1,49.9)
High consumption of red meat^‖,‡^	56.1 (49.4,62.9)	42.3 (38.0,46.6)	42.1 (36.4,47.7)	46.6 (43.2,50.0)
Harmful use of alcohol^¶,‡^	8.3 (3.7,13.0)	8.9 (6.7,11.0)	15.6 (11.4,19.7)	9.7 (7.8,11.7)
Physical inactivity^#^	10.2 (6.8,13.6)	11.7 (8.8,14.6)	16.4 (11.8,20.9)	11.9 (9.9,14.0)
N of risk factors**				
0	17.4 (13.8,21.0)	20.2 (17.4,23.0)	9.5 (7.3,11.7)	17.7 (15.8,19.6)
1	38.3 (31.8,44.9)	39.6 (35.3,43.9)	29.5 (25.0,34.0)	37.6 (34.5,40.8)
2	33.2 (25.4,41.0)	26.4 (21.9,31.0)	36.0 (30.0,42.0)	30.0 (26.4,33.7)
3+	11.1 (7.5,14.6)	13.7 (10.7,16.8)	25.0 (19.8,30.1)	14.6 (12.5,16.8)

*Percentages were weighted to represent the total population of Chongqing City. Numbers in parenthesis indicate 95% confidence intervals which took into account complex sample design of the survey.

^†^Using tobacco every day or on some days currently.

^‡^P value < 0.05 for trend test in prevalence across toothbrushing frequency.

^§^Daily Consumption of fresh fruit and vegetables <400 g.

^‖^Daily consumption of pork, mutton, or beef ≥100 g.

^¶^Daily consumption ≥25 g pure alcohol for men or ≥15 g women.

^#^<150 minutes of moderate activity or their metabolic equivalent per week.

**P value < 0.05 for correlation test between toothbrushing frequency and number of risk factors.

### Association between NCD BRF and toothbrushing

Table 3 showed the independent effect of number of BRF on the toothbrushing frequency, as well as the effects of other various covariates, as revealed by ordinal logistic regression. Tooth brushing frequency was independently associated with number of BRF an individual had. For instance, in respondents who had 3 BRF or more, the multi-variate adjusted cumulative odds of brushing teeth at a certain frequency or lower versus brushing more frequently were 2.1 (95% CI: 1.4–3.1) times higher than among those with no BRF. The adjusted COR was 1.5 (1.1–2.1) and 1.4 (1.0–1.8) for those who had two BRF and those who had one, respectively. Age, sex, education, annual household income, and residence location were also found associated with toothbrushing frequency. The adjusted COR increased steadily with age, while decreased with education and annual per capita household income. Men were 1.6 (1.1–2.2) times more likely to brush teeth less frequently than women. Rural respondents had 1.3 (1.0–1.7) times higher likelihood of brushing teeth less frequently than their urban counterparts. The estimated adjusted CORs for the number of behavioral risk factors are higher than crude CORs. This indicates that the association between tooth brushing and NCD behavioral risk factors is confounded by other demographic and socio-economic covariates.

**Table 3:** Effects of correlates on tooth brushing frequency in multiple ordinary logistic regression*.

Characteristics		Crude Cumulative Odds Ratio	Adjusted Cumulative Odds Ratio
Age (years)	18–29	Reference	Reference
	30–49	3.0 (1.7,5.4)	1.9 (1.0,3.7)
	50–69	6.3 (3.6,11.1)	3.1 (1.6,6.1)
	70+	14.0 (7.8,25.0)	5.6 (2.7,11.4)
Sex	Women	Reference	Reference
	Men	1.6 (1.2,2.0)	1.6 (1.2,2.2)
Education	Illiterate	17.3 (8.0,37.5)	7.9 (3.3,18.6)
	Primary school	9.9 (4.6,21.1)	5.5 (2.4,12.6)
	Junior high school	3.6 (1.6,8.0)	2.5 (1.1,5.8)
	Senior high school	3.0 (1.3,6.9)	2.5 (1.0,6.5)
	College graduate	Reference	Reference
Marital Status	Single	Reference	Reference
	Married or cohabiting	3.0 (1.3,7.0)	0.9 (0.4,2.5)
	Single-Separated/divorced/widowed/others	6.1 (2.5,14.4)	1.0 (0.4,2.6)
Employment	Unemployed	Reference	Reference
	Employed	1.3 (1.0,1.7)	1.0 (0.7,1.4)
Annual household income per capita (US$)^†^	Don’t know/not sure/refused	2.6 (1.7,3.7)	1.7 (1.1,2.6)
	1st quartile, <1058	5.2 (3.6,7.6)	2.3 (1.5,3.6)
	2nd quartile, 1059–1905	2.6 (1.3,5.1)	1.8 (1.1,3.0)
	3rd quartile, 1906–3704	1.9 (1.2,3.0)	1.7 (1.1,2.7)
	4th quartile, >3705	Reference	Reference
Self-report hypertension	No	Reference	Reference
	Yes	1.8 (1.4,2.3)	0.9 (0.7,1.2)
Self-report diabetes	No	Reference	Reference
	Yes	1.0 (0.7,1.5)	0.7 (0.4,1.1)
BMI	Normal	Reference	Reference
	Overweight	1.0 (0.8,1.3)	1.0 (0.8,1.4)
	Obesity	1.2 (0.6,2.4)	1.2 (0.6,2.5)
Number of behavioral risk factors	0	Reference	Reference
	1	1.1 (0.8,1.3)	1.4 (1.0,1.8)
	2	1.1 (0.8,1.6)	1.5 (1.1,2.1)
	3+	2.0 (1.4,2.8)	2.1 (1.4,3.1)
Residence location	Urban	Reference	Reference
	Rural	2.4 (1.9,3.2)	1.3 (1.0,1.7)

*Probabilities modeled are cumulated over the lower tooth brushing frequency. Numbers in parenthesis indicate 95% confidence intervals which took into account the complex sample design of the survey. ^†^Based on the exchange rate of 6.3 renminbi to US$ 1 that was in effect on 30 September 2012.

## Discussion

This study provided a snapshot of the relationship between toothbrushing frequency and NCDs related RFS among an adult population of China. We found NCDs BRF tended to cluster within respondents who brushed teeth less frequently. The association in between is statistically significant after adjusting for potential confounders. The findings yielded by this population-based study provide informative basis allowing to design proper public health program focusing on both oral health promotion and NCDs prevention.

Respondents who had poor oral hygiene practice often exposed to important NCDs related BRF, such as smoking, harmful drinking and insufficient intake of vegetable and fruit. More than half of the adults who rarely or never brushed their teeth reported their smoking when the survey was conducted. This percentage was surprisingly high as smoking prevalence among Chinese adults in 2013 was 27% for both genders and only 2.3% for women^18^. Epidemiological studies consistently revealed an association between smoking and periodontitis^14^, and had identified smoking as an important risk factor for oral cancer^15^. In addition, accumulative evidences indicated causal relationship between periodontal disease cardiovascular disease^5,6^ and diabetes^2,3^. Therefore, population with poor oral hygiene are at risk of developing NCDs and oral diseases. Harmful use of alcohol was also more common among population with lower toothbrushing frequency. Similarly, harmful use of alcohol was more frequent among the population with lower toothbrushing frequency, as previously reported in the literature^19^. For example, a cross-sectional study in Japan analyzed annual medical checkup data of 85,866 individuals and showed a much higher prevalence of heavy drinking among those who brushed their teeth less than once a day (47.1%) when compared to those who brushed after each meal (30.9%)^19^. The present study revealed correlations that had seldom been reported previously, such as correlation of toothbrushing frequency with consumption of red meat, as well as with consumption of vegetable and fruit. This could be explained intuitively by the possible higher socioeconomic status of individuals with better oral health behavior^20^. High socioeconomic status usually means greater purchasing power and better access to agricultural products. However, we failed to find significant link between physical activity and toothbrushing frequency which had been reported elsewhere. For instance, the Scottish Health Survey reported higher prevalence of physical inactivity among population who rarely or never brushed teeth^12^. Covariations among NCDs related BRF are prominent in China and have been well documented^16^.

We found statistically significant association between oral hygiene behaviors and NCDs behavioral risk factors. The possible mechanism behind this association could be that they both share similar socio-economic determinants. For example, previous nationwide analysis reported that more NCD behavioral risk factors were clustered within individuals with lower socio-economic status^16^; the present study also showed tooth brushing frequency was reversely associated with education attainment and income (Table 3). Although the association might be non-causal, this finding still has potential implications for further researches, policy maker or the general public. It would be of much interest, especially in situation where resources are limited, to explore the value of taking oral hygiene behavioral as a proxy metric or predictor for overall NCDs health status or as an identifier to screen out high-risk individuals in population-based NCDs program, as measurements for tooth brushing is much cheaper and easier to assess than those NCDs behavioral risk factors. In addition, the association between toothbrushing frequency and clustering of NCDs related BRF shed new light on integrated prevention for NCDs and oral diseases. The interrelation between toothbrushing behavior, smoking, alcohol consumption and unhealthy diet demonstrates the importance of adequate brushing habits in preventing NCDs. Furthermore, integrating oral care into prevention and control of NCDs is beneficial as they share the common risk factors^21^. In turn, a healthy lifestyle helps adopting sustainable proper brushing habits, decreasing the risk of periodontal inflammation and resulting in an improved quality of life^22^. Common lifestyle interventions have been proposed to reduce the burden of NCDs and oral diseases, by adopting a collaborative approach targeting all common risk factors simultaneously^23^. However, toothbrushing which is a key element of good oral health has been a neglected factor in reinforcing the control of NCDs in China. Few NCDs prevention programs include oral health components or interventions. Before adopting integrated strategy, studies on the impact of common risk factor interventions are still needed to validate their benefits in local settings.

The study reported in this article is exploring for the first time the correlation between toothbrushing behavior and NCDs related RFS among the Chinese adult population. With population representative data, our findings hold fine external validity and can be generalized in the study area. Although the random survey design and big sample size might strengthen our findings, this study still had some limitations. First, most of data used in the study were collected by questionnaire, which may lack reliability and suffer from recall bias. In particular, self-reported toothbrushing might be biased and affected by the perceived social desirability of the behavior, but existing evidences supported the reliability of toothbrushing frequency in epidemiological studies^24^. Second, only toothbrushing was measured in the survey when collecting information on oral hygiene behavior. Other oral care practices, like dental floss use and mouthwash, may need further examination. Third, cross-sectional nature of the study could only reveal the correlation or association between factors. Any causal inference should be further examined in cohort or intervention studies.

Significant associations exist between oral care behavior and NCDs related BRF. Integrated intervention strategy involving both oral health and NCDS related BRF may be beneficial for oral health improvement and for prevention of NCDs. In addition, there is a need for public health promotion and campaigns in China to increase the awareness among health care professionals and patients about the possible link between toothbrushing and NCDs related BRF.

## Method

### Data source

In the present study, we used data from the Chongqing Health Behavior and Disease Burden Survey 2012 that was conducted between April and October 2012. The survey aimed to collect population representative information on health-related lifestyle behaviors and main diseases affecting health in Chongqing, a southwest municipality of P. R. China. A multistage sampling scheme was adopted to obtain a representative sample, and it was elaborated in our previous publication^25^. A total of 4485 individuals with age over 18 years participated in the survey, representing 29.9 million adults in Chongqing. The overall response rate was 83%. Each respondent provided written informed consent before data collection. Questionnaire-based interviews were administrated by field workers who received unified training on survey contents and interview skills and passed qualification examination. The ethical review committee of Chongqing Medical University approved the survey protocols. The survey was carried out strictly in accordance with the approved guidelines.

After excluding 5 individuals who failed to provide information of toothbrushing frequency and 15 with other missing study variables, there were 4465 individuals remained in the final analysis. Data used in the present study are only available upon reasonable request.

### Measurement

Survey questionnaire collected information on demographic characteristics, socio-economic status, NCDs relevant BRF, NCDs status, as well as oral health behaviors. Specifically, questions on BRF, such as tobacco using, alcohol drinking, physical activity and diet, were mainly borrowed from the questionnaire of WHO STEP surveillance^26^ and then adapted for local use. Five dichotomized BRF were constructed and their definitions are as follows. Current smoking was defined as self-reported tobacco use every day or on some days during survey period. According to the Dietary Guidelines for Chinese Residents^27^, consuming ≥15 g/day of pure alcohol for women and ≥25 g/day for men were defined as harmful use of alcohol. Consuming less than 400 g/day of fruit and vegetable was considered as insufficient^26^. High consumption of red meat was defined as consuming ≥100 g/day of beef, pork, or mutton^28^. Physical inactivity was defined as less than 150 minutes of moderate-intensity activity per week, or equivalent^29^. Status of both hypertension and diabetes were defined as self-report condition diagnosed by health professionals at hospital of county-level or above. Toothbrushing frequency was measured by asking the respondent, “In a typical day of the last 12 months, how often did you brush your teeth?”. Provided answers were categorized into three groups: ≥ twice a day, once a day, and rarely or never.

Height and weight of each respondent were also objectively measured using unified measuring tape and weight scale with standard operational procedure, to compute body mass index (BMI, or weight in kg divided by height in meters squared). BMI was further categorized into three groups: normal (BMI < 24), overweight (25 ≤ BMI < 30), and obesity (BMI ≥ 30).

### Statistical analysis

We first described basic characteristics of the study population by toothbrushing frequency. We estimated population weighted prevalence and 95% confidence intervals (95% CI) of the five BRF by toothbrushing frequency. Test for linear trend in the prevalence across toothbrushing frequency were performed with logistic regression that included toothbrushing frequency as a continuous variable. The distribution of clustering (number) of NCDs BRF were also determined. We tested the correlation between number of NCDs BRF and toothbrushing frequency using Rao-scott chi-square test^30^. In multiple ordered logistic regressions^30^, we quantified the association (measured by adjusted cumulative odds ratios, COR) between toothbrushing frequency and number of NCDs BRF, with adjustment of demographic characteristics, socio-economic status, NCDs status and BMI. COR reflects the cumulative odds of brushing teeth at a certain or lower frequency versus brushing at higher frequency against the cumulative odds in the reference group. In other words, the COR represents the average effect of covariates on the cumulative odds of brushing teeth at a certain or lower frequency. We considered residence location, NCDs status and BMI as potential confounders and had them controlled in the regression model. This was mainly because notable discrepancy was reported previously of oral health and BRF prevalence between Chinese urban and rural residents^18,31^, and lifestyle modification would possibly occur once one was diagnosed with NCDs. For all independent variables, we also provided the crude CORs that were estimated from univariate logistic regressions. Categorizations of model covariates and their corresponding crude and adjusted CORs were shown in Table 3.

In inferential analysis (Tables 2 and 3), computation was weighted to obtain population representative estimates. The weights were the product of sampling weight, which was the reciprocal of the probability of a particular individual being selected, and a post-stratification factor that adjusted for age and sex in accordance with the 2010 census population estimates of Chongqing City. To account for the complex design of the survey, 95% CIs were estimated using Taylor’s series method^30^. All analysis was performed in SAS 9.4 (SAS Institute Inc., Cary, USA).

## Data Availability

The datasets analyzed during the current study are available from the corresponding author on reasonable request.
